# Global Distribution Patterns of Dark Matter Fungi in Cold Seep: A Metagenomic Meta-Analysis

**DOI:** 10.3390/jof11120878

**Published:** 2025-12-11

**Authors:** Zhi-Feng Zhang, Yi Jiang, Jian Mao

**Affiliations:** 1 Southern Marine Science and Engineering Guangdong Laboratory (Guangzhou), Guangzhou 511458, China; 2 Institute for Advanced Study, Shenzhen University, Shenzhen 518060, China

**Keywords:** cold seep, fungal community, metagenomics, biogeography, assembly patterns

## Abstract

Deep-sea cold seep ecosystems, known for their unique geochemical niches and chemosynthetic communities, harbor numerous “dark matter fungi (DMF)” that remain understudied compared to their bacterial and archaeal counterparts. Integrating 105 metagenomic datasets from 12 global cold seep sites, this study systematically elucidated the diversity, biogeography, and assembly mechanisms of cold seep fungal communities. Our analysis revealed highly diverse and abundant fungi, spanning 21 phyla and 928 genera, characterized by the absolute dominance of Ascomycota and a pervasive presence of unclassified DMF. Crucially, the fungal communities exhibited significant geographical and seep-type heterogeneity, with peak abundance notably in methane seep environments. Further analyses revealed that fungal community diversity and structure are influenced by both spatial and biological factors. Moreover, assembly exhibits multi-scale characteristics: dispersal limitation is the primary determinant globally, while local-scale structure is jointly driven by environmental variables and biological interactions with key chemosynthetic genes. These findings establish the macro-biogeographical pattern of deep-sea cold seep fungi, underscoring their tight coupling with core energy metabolism and providing essential data for future research and potential utilization.

## 1. Introduction

Deep-sea cold seeps are distinctive ecosystems prevalent across continental margins, mid-ocean ridges, and active plate boundaries. They are characterized by the continuous expulsion of fluids enriched in chemical substances, notably methane and hydrogen sulfide [1,2,3]. These compounds serve as the primary energy source for highly productive chemosynthetic communities, which comprise free-living bacteria and archaea, alongside symbiotic invertebrates such as tubeworms, mussels, and clams [4,5,6,7]. Due to their intense chemosynthetic activity, which provides abundant energy and organic matter for associated microorganisms, including fungi, cold seep areas stand in stark contrast to the surrounding “deep-sea desert” and are thus often referred to as “deep-sea oases” [8,9,10].

Elucidating the global distribution patterns of cold seep microbiota is fundamental not only to understanding deep-sea ecology but also critical for prospecting novel enzymes and bioactive compounds with potential biotechnological applications [11]. Traditionally, our understanding of these communities was hindered by the limitations of culture-dependent methods, as >99% of marine microorganisms remain uncultured [12,13,14]. However, the advent of high-throughput sequencing and metagenomics has revolutionized this field, enabling the recovery of “microbial dark matter” and providing holistic insights into the metabolic potential and ecological roles of deep-sea microbiomes without the need for cultivation [15,16].

The unique geochemical environment of deep-sea cold seeps has made their microbial communities a focal point of intense research [11]. The key biogeochemical cycle in cold seep sediments is the anaerobic oxidation of methane (AOM), primarily catalyzed by anaerobic methane-oxidizing archaea (ANME) in syntrophic partnership with sulfate-reducing bacteria (SRB). This critical process not only consumes substantial quantities of the greenhouse gas methane but also serves as the main source of energy and carbon for the entire cold seep ecosystem [7,17,18]. Studies indicate that the composition of these microbial communities can vary significantly across different cold seep sites [11,19]. For example, the microbial biofilms at some seep sites are completely dominated by sulfur-oxidizing bacteria, while others exhibit dominance by ANME-1b archaea [18,20]. Furthermore, research suggests that cold seep eruption events can drive distinct shifts in microbial communities, consequently impacting carbon cycling [7,21].

Although fungi are known to play a crucial role in marine energy flow and element cycling [22,23], research specifically on deep-sea cold seep fungi remains relatively limited compared to that focused on bacteria and archaea. The majority of existing studies involving these communities primarily rely on overall analysis of eukaryotic communities based on ITS (internal transcribed spacer) regions [24,25,26,27,28,29,30,31,32,33]. Consequently, dedicated investigations into the diversity, community structure, and ecological functions of cold seep fungi are markedly scarce.

Nevertheless, the limited available research has documented a high abundance and diversity of fungi within cold seep sediments, predominantly encompassing the phyla Ascomycota, Basidiomycota, and Chytridiomycota [26,30,33]. While many of these fungi resemble species found in terrestrial or other marine environments, a substantial fraction comprises unique and unclassified fungal groups, frequently termed “dark matter fungi” (DMF), which persistently inhabit deep-sea cold seeps [26,28,29]. Regarding their ecological functions, fungi in cold seeps are hypothesized to act primarily as decomposers, actively participating in the degradation of complex organic matter [26,34]. Furthermore, they may also facilitate hydrocarbon degradation and methane utilization, potentially by secreting hydrophobic proteins that aid in methane dissolution [24,26,32]. Additionally, some fungi could function as parasites or pathogens, thus potentially impacting the stability of cold seep faunal communities [25,35,36]. Further analyses based on ITS or 18S fragments further indicate that environmental factors such as methane seepage rate, organic matter content, and oxygen availability exert a significant influence on the structure of these fungal communities [30,33,37].

As previously established, although existing studies have touched upon the spatial distribution and influencing factors of cold seep fungi, our comprehensive knowledge regarding their diversity, community structure, underlying assembly mechanisms, and global biogeographical patterns remains severely limited. Compounding this issue, the inconsistencies and inherent biases stemming from the use of varied marker genes and sequencing methodologies in previous research have often provided only restricted and fragmented insights into these fungal communities. Given the unique environmental characteristics of cold seep ecosystems, we hypothesize that cold seep fungal communities exhibit a non-random distribution, co-determined by dispersal limitation (global scale) and metabolic coupling with chemoautotrophic prokaryotes (local scale). To address this hypothesis, this study utilized 105 metagenomic datasets from 12 global stations encompassing five distinct cold seep types to thoroughly investigate the associated fungal diversity and community structure. A key innovation of this research is the identification of factors influencing their global distribution, including previously overlooked biological variables such as bacterial diversity and autotrophic carbon fixation capacity. By adopting this comprehensive approach, this research aims to bridge the existing knowledge gap and significantly deepen our mechanistic understanding of the role of fungi in extreme deep-sea ecosystems.

## 2. Materials and Methods

### 2.1. Data Collection

**Figure 1:**
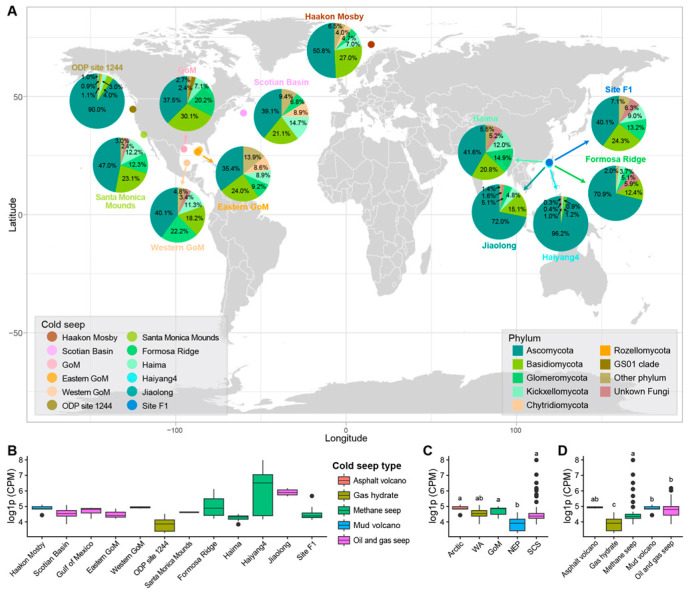
Distribution and characteristics of fungal communities in cold seep ecosystems. (**A**) Fungal community structure at each cold seep site. (**B**–**D**) Comparison of fungal abundance stratified by (**B**) specific cold seep sites, (**C**) major geographic locations, and (**D**) cold seep types. The full names of the geographic abbreviations in subplot D can be found in the Materials and Methods section. Boxplots in subplot (**B**–**D**) illustrate the median values (middle line), interquartile range (box boundaries), and 1.5 times the interquartile range (whiskers). The letters above the boxplots represent the results of the significance analysis; different letters indicate statistically significant differences.

By searching and screening all published papers involving cold seep ecosystems and metagenomic sequencing using Google Scholar, a preliminary collection of 109 publicly available raw metagenomic datasets from cold seep sediments worldwide was compiled [11]. This initial dataset spanned 12 sites representing five distinct cold seep types globally (Figure 1A). We then refined this collection by excluding four samples due to either low sequencing yield or data redundancy, yielding a final, high-quality dataset of 105 metagenomic datasets. These sediment samples originated primarily from the South China Sea (SCS, 66 samples), followed by the Northeast Pacific (NEP, 15 samples), the Gulf of Mexico (GoM, 11 samples), the Western Atlantic (WA, 7 samples), and the Arctic Ocean (6 samples). Furthermore, the samples collectively covered five major seep categories: methane seep (62 samples), oil and gas seep (19 samples), gas hydrate (15 samples), mud volcano (6 samples), and asphalt volcano (3 samples). All datasets were retrieved from the NCBI Sequence Read Archive (SRA) using SRA-Toolkit V3.3.0 (https://github.com/ncbi/sra-tools (accessed on 15 August 2025)), and their relevant information was annotated in Table S1.

Concurrently, the spatial location and physicochemical data for each sampling site were collected for subsequent analysis of influencing factors. It is noteworthy that different studies, or cold seep sampling campaigns, measured distinct sets of physicochemical parameters. For clarity, the variations in data collection across different cold seep sites are summarized. In the SCS, ten samples from Site F1 were analyzed for concentrations of methane, TN (total nitrogen), TOC (total organic carbon), and SO_4_^2−^. Additionally, twenty-four samples from the Haima, Jiaolong, and Haiyang 4 seeps focused specifically on TN and TOC content. In the NEP, samples from ODP Site 1244 were measured for TC, TN, TS, TIC, TOC, and CaCO_3_ levels. Finally, six samples from the Haakon Mosby Mud Volcano in the Arctic were characterized by parameters including SO_4_^2−^, H_2_S, DIC (dissolved inorganic carbon), alkalinity, NH_4_^+^, NO_3_^−^, methane oxidation rate (MOR), and sulfate reduction rate (SRR).

### 2.2. Microbial Diversity and Function Analysis

The metagenomic raw reads were first quality-trimmed using Sickle v1.33 software with default parameters (https://github.com/najoshi/sickle (accessed on 15 August 2025)). The fungal community profiles were generated directly from the trimmed metagenomic reads. Specifically, ITS gene fragments were predicted and annotated by searching the raw metagenomic reads against the full UNITE+INSD database for fungi v10.0 [38] using BLASTn search [39]. The fungal sequences identified via BLASTn were subsequently clustered and taxonomically annotated at the species level. The relative abundance of each species was then quantified using CPM (Counts Per Million) values, which represent the frequency of that species per million total reads in each metagenomic dataset. Specifically, the CPM value was calculated as:

CPM = (Read count of taxa/Total quality filtered reads in sample) × 10^6^

The use of CPM values is a reliable and unbiased method for estimating the relative abundance of fungi [40]. Sequences that could not be accurately annotated to the species or genus level were collectively designated as DMF, and their proportion and distribution were analyzed within higher taxonomic levels. Subsequently, α-diversity indices, including OTU richness, Shannon index, and Simpson index, were calculated using the vegan package [41] and species table. To investigate the fungal taxa that are stably present and universally contributing across global cold seeps, we screened for fungal genera present in 80% of the samples as the core mycobiota [42,43].

Similarly, the abundance of prokaryotic microorganisms was quantified by counting the number of 16S rRNA genes identified via a BLASTn search against the SILVA database V138.1 [44], and their relative abundance was also calculated as CPM values across the samples. Our analysis focused primarily on ANME and SRB, as these groups are crucial primary producers in cold seep environments and may significantly influence the local fungal community [10]. Based on published literature [9,45,46], these key functional groups were filtered from the 16S annotation results to calculate their respective CPM values in each metagenomic dataset.

In cold seeps, ANME and SRB catalyze methane oxidation and sulfate reduction, respectively, via enzymes encoded by key functional genes such as *mcr* (methyl coenzyme M reductase) and *dsr* (dissimilatory sulfite reductase). They further produce organic matter by fixing CO_2_, primarily utilizing the Wood–Ljungdahl (WL) pathway [45,47]. To quantify this metabolic potential, we analyzed the abundance of key functional genes related to these pathways in the metagenomic datasets: The *mcr* and *dsr* gene sequences were downloaded from the UniProt database. We then performed a search for potential *dsr* and *mcr* functional genes using DIAMOND blastx with stringent parameters “-e 1e-10 --id 0.5” [48]. The key metabolic genes for autotrophic carbon fixation pathways were identified using the GhostKOALA [49] and the Kyoto Encyclopedia of Genes and Genomes (KEGG) database [50]. Finally, the CPM value was calculated for each identified functional gene within every metagenomic dataset to estimate its relative abundance.

### 2.3. Statistical Analyses

The variations and significant differences in fungal abundance and diversity across different cold seep sites and types were first assessed using a one-way ANOVA followed by Tukey’s honest significant difference (HSD) post hoc test [51]. Subsequently, Linear discriminant analysis Effect Size (LEfSe) was employed to identify fungal biomarkers characterizing different cold seep types and geographical regions [52]. A threshold of linear discriminant analysis (LDA) score > 2.5 and *p* < 0.05 was used to determine statistical significance. Then, we calculated the Pearson’s correlation coefficients and their corresponding *p*-values between fungal abundance and a comprehensive variables dataset. These variables included environmental factors (e.g., seawater depth, sediment depth, CH_4_, TC, TN, TS, TOC, SO_4_^2−^, and H_2_S) and biological factors (e.g., abundance of bacteria, ANME, and SRB, and abundance of *mcr*, *dsr*, and carbon fixation genes). This step was critical for identifying factors significantly correlated with fungal abundance.

To explore the integrated influence of environmental and biological factors on fungal community structure, we constructed a species-level fungal abundance table. The abundance table was first subjected to Hellinger transformation, after which the dissimilarity between samples was calculated using the Bray–Curtis method to construct a distance matrix [41]. The resulting distance matrix was then used for community ordination via Nonmetric Multidimensional Scaling (NMDS), performed using the metaMDS command in the vegan package in R language. Subsequently, we employed several analyses to decompose the influence of variables on the community structure. Firstly, the envfit function in the vegan package was used to preliminarily investigate the influence of environmental and biological factors on the fungal community (based on 999 permutations). Permutational multivariate analysis of variance (PERMANOVA) was then employed to test the community similarity of different sample groups. Finally, a distance-based redundancy analysis (db-RDA) using Bray–Curtis dissimilarities was performed to explore the variations explained by environmental and biological variables [53].

## 3. Results

### 3.1. Distribution and Abundance of Fungal Taxa in Cold Seeps

A total of 1,125,492 fungal ITS sequences were successfully obtained from the compiled metagenomic datasets. However, due to substantial variations in sequencing depth across the included studies, the raw ITS sequence counts varied drastically between datasets, ranging from a low of 254 (SRR5716311) to a high of 97,111 (SRR19020587). To ensure comparability, the ITS sequence counts for each sample were processed by CPM normalization. Even after normalization, the resulting CPM values continued to show considerable inter-sample variation. The lowest observed CPM values were 25.63 (SRR5716310) and 25.67 (SRR5716311). These samples originated from gas hydrates at the ODP site 1244 cold seep (Northeast Pacific) at sediment depths of 20.69 m and 68.55 m, respectively. In sharp contrast, two near-surface sediment samples (0.02 m and 0.1 m) from the Haiyang4 methane seep in SCS displayed significantly higher CPM values, reaching 1147.4 (SRR19020584) and 2938.73 (SRR19020587).

Furthermore, obvious heterogeneity was observed in fungal abundance across different cold seep sites (Figure 1B). The lowest fungal abundance was observed at ODP Site 1244 in the Northeast Pacific, with a mean CPM value of 48.85 ± 22.04. In contrast, multiple cold seeps in the SCS—specifically Haiyang4, Formosa Ridge, and Jiaolong—all exhibited mean fungal CPM values exceeding 200, reaching 980.81 ± 1184.07, 373.62 ± 88.37, and 200.12 ± 176.18, respectively. Notably, the Haiyang4 cold seep has three samples with fungal CPM values greater than 500 (674.4, 1147.4, and 2938.73).

Geographically, the cold seeps in the SCS displayed the highest overall fungal abundance, with a mean ITS CPM value of 174.41 ± 385.37, potentially driven by the exceptionally high abundance at the Haiyang4 seep (Figure 1C). It was followed by the Arctic (129.03 ± 27.80), the GoM (129.03 ± 27.80), and the WA (112.45 ± 29.81). The region with the lowest abundance was the NEP (52.30 ± 25.10). Regarding cold seep types, the highest fungal abundance was found in methane seeps (161.56 ± 393.81) and oil and gas seeps (157.75 ± 123.29), whereas the lowest was in Gas hydrate environments (52.30 ± 25.10) (Figure 1D).

From the perspective of fungal diversity, the cold seeps in the Eastern GoM (273.00 ± 125.53) and the Scotian Basin (227.71 ± 123.59) exhibited the highest species richness. Conversely, the cold seep at ODP Site 1244 showed the lowest species richness (26.07 ± 20.05). Regarding geographical distribution, cold seeps located in the Western Atlantic displayed the highest fungal species richness (227.71 ± 123.59), while those in the Northeast Pacific had the lowest (32.80 ± 32.44). In terms of seep type, Oil and gas seeps and Gas hydrate seeps demonstrated the highest and lowest fungal species richness, respectively (Figure S1).

The annotation results revealed that the fungal ITS sequences obtained belong to 21 phyla, 71 classes, and 928 genera. Ascomycota emerged as the overwhelmingly dominant phylum, exhibiting the highest mean CPM (94.40) and accounting for over half of the total community relative abundance (51.30%). The remaining abundant phyla, in descending order of abundance, were: Basidiomycota (mean CPM: 19.73; 19.30%), Glomeromycota (mean CPM: 10.60; 10.92%), Kickxellomycota (mean CPM: 8.33; 9.21%), and Chytridiomycota (mean CPM: 2.76; 2.61%). All other detected phyla, such as Rozellomycota, GS01 clade, Blastocladiomycota, Mucoromycota, and Monoblepharomycota, had a relative abundance below 1%.

Although Ascomycota, Basidiomycota, and Glomeromycota collectively formed the dominant groups in most investigated cold seeps, their relative abundances displayed obvious site-to-site variation (Figure 1A). For instance, the relative abundances of Ascomycota ranged narrowly from 35.4–40.1% in three cold seeps located in the GoM, with Basidiomycota and Glomeromycota from 18.2–30.1%, and 9.2–22.2%, respectively. In contrast, five cold seeps in SCS exhibited much broader ranges: 40.1–96.2% for Ascomycota, 1.2–20.8% for Basidiomycota, and 0.9–14.9% for Glomeromycota. Notably, the relative abundance of Ascomycota exceeded 90% in specific locations, the Haiyang4 cold seep in SCS and the ODP Site 1244 cold seep in NEP. Furthermore, the CPM for Ascomycota in the Haiyang4 cold seep was exceptionally high, ranging from 1108.87 to 2906.13, reinforcing its site-specific dominance.

**Figure 2:**
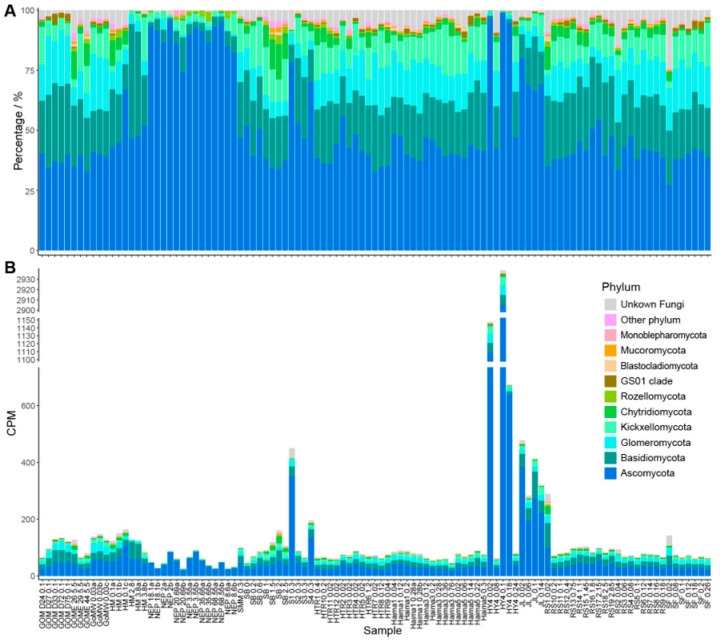
Fungal community profiles at the phylum level in global cold seeps. (**A**) Relative abundance of the top 10 most abundant fungal phyla across all samples. (**B**) Absolute abundance of the top 10 most abundant fungal phyla across all samples. GOM, Gulf of Mexico; GOME, Eastern Gulf of Mexico; GoMW, Western Gulf of Mexico; HM, Haakon Mosby mud volcano; NEP, Northeast Pacific ODP site 1244; SM, Pacific Ocean Santa Monica Mounds; SB, Scotian Basin; S, Formosa Ridge; HTR, Haima cold seep; HY, Haiyang4 cold seep; JL, Jiaolong cold seep; RS and SF, Site F1 cold seep.

At the genus level, the most abundant genera based on mean CPM were *Aspergillus* (17.93), *Orphella* (8.10), *Candida* (5.79), *Rhizophagus* (2.35), and *Sebacina* (2.15). In terms of relative abundance, the top genera were *Aspergillus* (18.96%), *Candida* (9.39%), *Orphella* (8.99%), *Sebacina* (2.55%), and *Rhizophagus* (2.39%). Other genera with relative abundances exceeding 1% included *Diaporthe* (1.55%), *Microbotryum* (1.42%), *Phlegmacium* (1.22%), and *Tirmania* (1.12%) (Figure 2). A notable finding is the extremely high abundance of an unclassified fungus belonging to the family *Pyronemataceae*. This unclassified taxon exhibited an average CPM of 58.85 and an average relative abundance of 10.48%, positioning it as one of the most dominant groups overall (Figure 2). The high abundance of *Pyronemataceae* appears to be primarily driven by samples from the Jiaolong and Haiyang4 cold seeps in SCS, where the average CPM reached 917.85 and 217.56, and the average relative abundance was 60.20% and 57.07%, respectively. When samples from these two sites were statistically excluded, the average CPM of *Pyronemataceae* dropped to 7.50, and the average relative abundance fell to 5.94%.

The core mycobiota of the cold seep ecosystem, defined as the fungal species present in 80% of all samples, consisted of 11 fungal genera (*Amanita*, *Aspergillus*, *Candida*, *Coltricia*, *Craterellus*, *Inocybe*, *Orphella*, *Phlegmacium*, *Rhizophagus*, *Ruinenia*, and *Sebacina*). Among these, *Aspergillus* and *Orphella* were detected in all samples, making them the most widespread fungal genera in cold seeps. They were also the genera with the highest relative abundance, reaching 18.96% and 8.99%, respectively. Concurrently, we identified 10 core fungal groups whose genus-level taxonomy remains unknown, with the family *Pyronemataceae* showing the highest average relative abundance at 10.47%.

Further LEfSe analysis revealed distinct fungal features differentiating the investigated environments (Figure S2). Across the geographic locations, 127 significantly differential biomarker genera (LDA > 2.5) were identified. Notably, nine genera exhibited highly specific enrichment with LDA > 4. *Hyphodontia* and *Sparassis* characterized the Arctic; *Orphella* was specific to the Western Atlantic; the Gulf of Mexico was marked by *Rhizophagus* and *Microbotryum*; *Candida* and *Tirmania* defined the Northeast Pacific; and the South China Sea was distinguished by *Aspergillus* and *Sebacina*. Furthermore, when categorized by cold seep type, 80 significantly differential biomarker genera (LDA > 2.5) were determined. Ten genera showed strong discriminatory power with LDA > 4. *Aspergillus*, *Orphella*, *Microbotryum*, and *Rhizophagus* were biomarkers for the Asphalt volcano type; *Candida* and *Tirmania* for the Gas hydrate type; *Sebacina* for the Methane seep; *Hyphodontia* and *Sparassis* for the Mud volcano type; and *Diaporthe* was specific to the Oil and gas seep type.

### 3.2. Assembly Patterns of the Fungal Community in Cold Seeps

**Figure 3:**
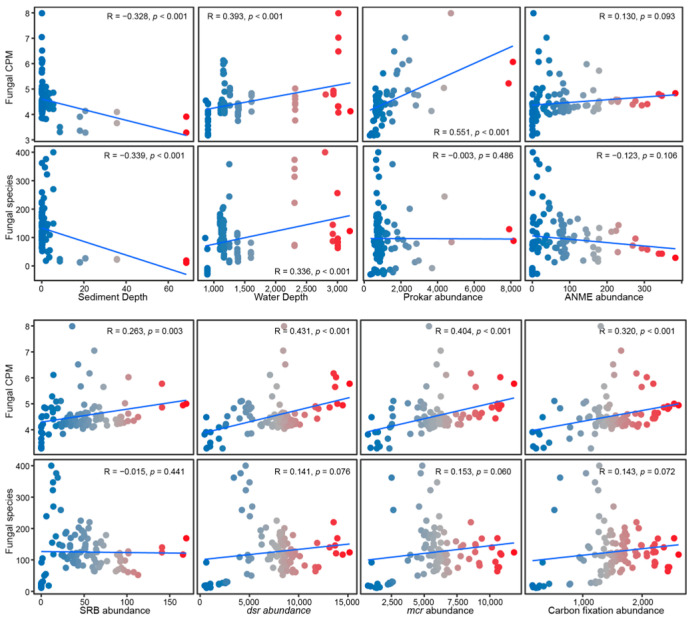
Correlations of fungal diversity and abundance to spatial and biotic parameters. Spatial factors: sediment depth and water depth. Biotic factors: prokaryotic abundance, ANME abundance, SRB abundance, and marker gene abundances (*dsr*, *mcr*, and carbon fixation genes). The correlation coefficient (R) and *p*-values are presented within the graph.

Across all global cold seeps, both fungal diversity and abundance exhibit a significant negative correlation with sediment depth, but a significant positive correlation with water depth (*p* ≤ 0.001). Furthermore, biotic factors such as the abundance of prokaryotes, SRB, *dsr* gene, *mcr* gene, and carbon fixation gene demonstrated a strong positive relationship with fungal abundance. Conversely, fungal species diversity showed no significant correlation with any of these measured biotic factors (Figure 3, Table 1), suggesting that the abundance of microorganisms involved in the core metabolism is the key determinant of fungal abundance. Regionally, fungal diversity in GOM was significantly negatively correlated with several biotic factors (ANME, *dsr*, *mcr*, and carbon fixation gene), whereas abundance therein displayed a significant positive correlation with most of these biotic factors (*p* ≤ 0.05). In the Northeast Pacific, both diversity and abundance are significantly positively correlated with most biotic factors (*p* ≤ 0.05). In SCS, abundance exhibited a significant positive correlation with water depth (*p* ≤ 0.05) and SO_4_^2−^ (*p* ≤ 0.001); However, abundance was also significantly negatively correlated with CH_4_ (*p* ≤ 0.01). In the Arctic region, both diversity and abundance showed a strong negative correlation with sediment depth. Additionally, diversity was significantly negatively correlated with *dsr*, *mcr*, and the carbon fixation gene (*p* ≤ 0.05). In the WA, both diversity and abundance were significantly positively correlated with sediment depth (*p* ≤ 0.05) (Table 1).

**Figure 4:**
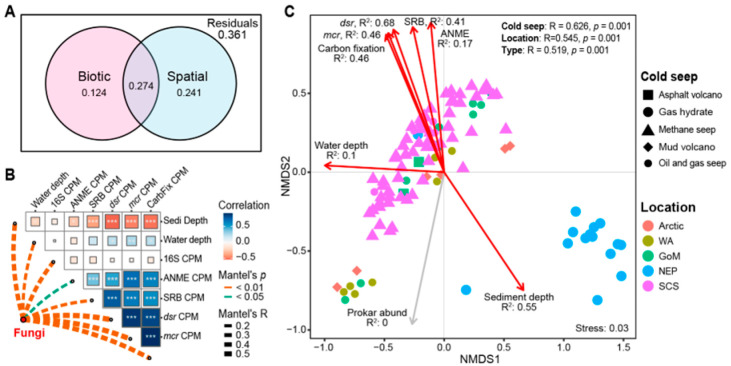
Effects of spatial and biotic selection on fungal community structure. (**A**) Variation partition analysis (VPA) based on Bray–Curtis dissimilarity matrices partitioning the relative contributions of spatial and biotic factors to the total variation in fungal community structure. (**B**) Effects of spatial and biotic variables on fungal community based on the Mantel test. The top right heatmap displays the inter-variable correlations among the spatial and biotic variables, where “*, **, and ***” denote *p* ≤ 0.05, 0.01, and 0.001, respectively. The dashed lines linking the red dots and heatmaps represent the influences of individual variables on fungal community structure. Line width indicates the effect magnitude, and line color indicates the statistical significance (*p*-value). (**C**) Nonmetric Multidimensional Scaling (NMDS) ordination of fungal community compositions. Similarity values among samples grouped by cold seep, geographical location, and seep types are examined via the analysis of similarities (ANOSIM) and shown in the upper right. The contribution of spatial and biotic factors to fungal community variation is indicated by the R-squared value and length of the arrows. Red arrows signify a statistically significant correlation, while gray arrows represent a non-significant correlation.

Variation partition analysis (VPA) revealed that spatial and biotic factors collectively explain 73.9% of the variation in the fungal community structure, despite the lack of physicochemical parameter data. Specifically, spatial factors explained 51.5% of the variation, biotic factors accounted for 39.8%, and their shared explanatory proportion was 27.4% (Figure 4A). As indicated by the correlation analysis results, this substantial overlap is likely due to the co-occurrence relationships among the explanatory variables (Figure 4B). Factor correlation analysis showed a strong and significant positive correlation among the abundances of ANME, SRB, *dsr* gene, and *mcr* gene. Water depth exhibited a significant, though weaker, positive correlation with the abundances of *dsr*, *mcr*, and the carbon fixation gene. Conversely, sediment depth exhibited a strong and significant negative correlation with the biotic factors, suggesting that the abundance of these key microbial and metabolic components decreases markedly with increasing sediment depth (Figure 4B). Mantel’s test demonstrated that the fungal community structure was significantly influenced by a broad range of both spatial and biotic factors: water depth (Mantel’s R = 0.199, *p* = 0.003), sediment depth (Mantel’s R = 0.400, *p* = 0.001), abundance of prokaryotes (Mantel’s R = 0.364, *p* = 0.001), ANME (Mantel’s R = 0.150, *p* = 0.01), SRB (Mantel’s R = 0.214, *p* = 0.001), *dsr* gene (Mantel’s R = 0.577, *p* = 0.001), *mcr* gene (Mantel’s R = 0.547, *p* = 0.001), and carbon fixation gene (Mantel’s R = 0.449, *p* = 0.001).

**Figure 5:**
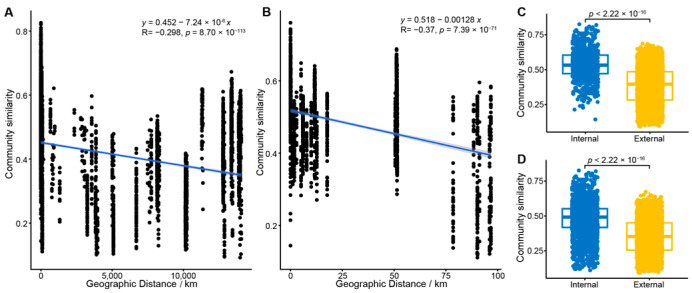
Distance-decay patterns and geographic control over fungal community similarity. (**A**,**B**) Distance-decay patterns of fungal community similarity in (**A**) global and (**B**) South China Sea cold seeps. The shaded areas represent the 95% confidence interval of the fitted regression lines. The fitted regression formula, the associated correlation coefficient (R), and the *p*-value are shown in each panel. (**C**,**D**) Comparison of community similarity between on-seep (internal) and off-seep (external) samples for (**C**) the South China Sea region and (**D**) all global cold seep sites.

The Nonmetric Multidimensional Scaling (NMDS) ordination clearly revealed that fungal communities clustered distinctly based on the cold seep’s geographical location and type, strongly suggesting a higher similarity among fungal communities originating from the same location and seep type (Figure 4C). This structural pattern was robustly supported by a subsequent PERMANOVA analysis, which demonstrated that both geographical location (R = 0.545, *p* = 0.001) and cold seep type (R = 0.519, *p* = 0.001) exerted a strong and significant influence on the overall fungal community composition (Figure 4C). Furthermore, both the PERMANOVA and community similarity analyses consistently indicated a greater similarity among fungal communities sampled within the same cold seep (Figure 4C and Figure 5A).

Consistent with Mantel’s test, the envfit analysis identified that the spatial factors (water depth and sediment depth) and all tested biological factors had a significant impact on the fungal community (*p* = 0.001). Among these variables, *dsr* gene abundance (R^2^ = 0.68) and sediment depth (R^2^ = 0.55) were the most influential drivers, whereas water depth had the smallest effect (R^2^ = 0.1). Regarding spatial distribution, the Bray–Curtis similarity of the fungal communities was significantly negatively correlated with increasing geographical distance (*p* < 0.001), establishing a prominent distance-decay relationship (Figure 5). This pattern was also evident within the SCS cold seep area, which accounts for the largest number of sites and samples (Figure 5B). However, the community similarity within the SCS region was higher compared to the global-scale similarity (Figure 5).

Aligning with these observations, we observed that fungal community similarity within an individual cold seep was significantly higher than the similarity of communities sampled outside the seep boundaries. Similarly, fungal communities from cold seeps in the same geographical region exhibited higher similarity than those from different geographical regions (*p* < 0.001) (Figure 5C,D). Collectively, these findings strongly suggest that the assembly pattern of the fungal community is distinctly shaped by local conditions, resulting in clear characteristics both across different global locations and within individual cold seep environments.

## 4. Discussion

By integrating 105 metagenomic datasets from 12 cold seep sites globally, this study systematically delineated the diversity, biogeography, and community assembly mechanisms of deep-sea cold seep fungi. Our findings not only confirm that these unique environments harbor abundant, diverse, and largely uncharacterized fungal taxa, but also determined that their community structure is fundamentally co-driven by spatial factors, local environmental conditions, and critical biological interactions with core microbial metabolisms.

### 4.1. Diversity and Unique Global Distribution Pattern of Cold Seep Fungal Community

Documenting fungal taxa across 21 phyla and 928 genera, this study highlights the pronounced diversity of cold seep fungal communities. Ascomycota emerged as the predominant phylum in global cold seeps (mean relative abundance: 51.30%), followed by Basidiomycota and Glomeromycota [24,26,28,30,54,55]. The consistent dominance of Ascomycota across most cold seep types and geographical regions underscores its widespread adaptability to extreme deep-sea conditions, such as high pressure and low temperature [24,26,27]. The identification of 21 fungal genera and associated unclassified taxa ubiquitously present across all cold seep samples strongly suggests their critical functional significance. The prevalence of known saprophytic genera, including *Aspergillus*, *Candida*, and *Inocybe*, points toward the core mycobiota’s primary function in the degradation of complex allochthonous organic carbon (e.g., lignin and cellulose) [56]. This process of organic matter remineralization is critical, as the fungi collaborate with the methane-rich anaerobic prokaryotes (e.g., anaerobic methanotrophs and sulfate-reducing bacteria) to collectively regulate the benthic carbon cycle in the sediments [57,58]. The high abundance and confirmed connectivity of *Aspergillus* within environmental fungal networks make its contribution particularly noteworthy. Moreover, the presence of typically symbiotic genera such as *Rhizophagus* and *Sebacina* hints at their potential involvement in unidentified trophic interactions or symbiotic relationships, which would provide supplementary energy and nutritional support to the cold seep food web [59,60]. Collectively, the core mycobiota provides an indispensable contribution to maintaining the material flux and ecological stability of the cold seep environment.

Conversely, fungal abundance exhibited significant spatial and environmental heterogeneity. The highest fungal abundance (mean CPM: 174.41 ± 385.37) was found in SCS, greatly surpassing the lowest abundance observed at the ODP Site 1244 in the NEP (mean CPM: 48.85 ± 22.04). This disparity was similarly evident across cold seep types, with methane seep and oil/gas seep environments hosting significantly higher fungal abundance than gas hydrate areas (Figure 1D). This striking pattern is likely due to the differential supply of energy and carbon resources provided by seep fluids (e.g., methane flux and complex hydrocarbons) [7,19]. Specifically, the concentrated input of chemical substances like methane and hydrogen sulfide supports active chemosynthetic primary production, which, in turn, supplies sufficient energy and organic carbon sources for fungi acting as decomposers, thereby driving the observed regional abundance variation [33,34,47]. Concurrently, the distinct fungal biomarker patterns identified are highly indicative of a rigorous metabolic selection imposed by the unique geochemical constraints of each cold seep niche. The pronounced enrichment of the ubiquitous *Aspergillus* and the yeast *Candida* at hydrocarbon-dominated sites, specifically Asphalt volcanoes and Gas hydrates, underscores their critical role in hydrocarbon catabolism [61]. Conversely, the dominance of lignocellulose-degrading Basidiomycetes, exemplified by *Hyphodontia* and *Sparassis*, in Arctic/Mud volcano environments signifies a distinct functional specialization for the mobilization of refractory, deep-seated organic carbon ejected from the subsurface [32]. Collectively, these biomarkers reveal a clear functional partitioning within the core mycobiota, demonstrating adaptation to exploit the specific energy subsidies defining each cold seep habitat. Meanwhile, distinct fungal taxa correlate with specific seep types, suggesting a degree of functional specialization [62]. However, the widespread distribution of “core” taxa (e.g., *Aspergillus*) points towards functional redundancy, where diverse taxonomical groups may perform similar ecological roles (e.g., generalist decomposition) to maintain ecosystem stability under fluctuating seep intensities [63].

A notable finding in this study is the pervasive presence of DMF, whose unannotated sequences constituted a substantial proportion of the total fungal abundance [26]. Specifically, unclassified fungi belonging to *Pyronemataceae* had a mean relative abundance of 10.48%, with peak concentrations observed at the Jiaolong and Haiyang4 cold seeps in SCS, where average CPMs reached 917.85 and 217.56, respectively. This exceptional local dominance may be attributed to their strong degradation capability, highly efficient nutrient absorption, and diverse ecological functions [64,65], allowing them to thrive in high-pressure, low-temperature, and chemically reducing environments. Consistently, previous metagenomic studies have detected a large number of lignin-degrading enzymes and various other hydrocarbon-oxidizing enzyme genes derived from fungi in cold seep microbial communities [32]. Collectively, these DMFs likely represent unique and endemic lineages adapted to these challenging conditions. Meanwhile, their pervasive presence underscores the vast reservoir of unknown fungal taxa and their critical functions awaiting exploration in deep-sea environments [66,67,68].

### 4.2. Multi-Scale Assembly Model of Cold Seep Fungal Communities

Understanding the forces that shape community composition and structure remains a major goal of microbial ecology [69,70,71]. In nature, the formation and succession of biological communities are simultaneously governed by deterministic and stochastic factors. The former, often termed homogeneous selection, includes constraints imposed by both abiotic (environmental factors) and biotic (inter-species interactions) factors, while the latter encompasses unpredictable ecological events, such as limited dispersal, birth, death, and migration [69,70]. Given that microbial chemoautotrophy is the primary energy source in cold seeps and a main driver of community succession, this study incorporates key biological factors, such as microbial abundance and the abundance of chemoautotrophic carbon fixation genes, into the fungal community structure analysis, revealing that the assembly of cold seep fungal communities is co-driven by multidimensional factors.

Deterministic processes: Deterministic processes play the core role in determining the assembly of cold seep fungal communities. VPA revealed that the variation in the fungal community is primarily co-shaped by spatial factors (51.5% independently) and biological factors (39.8% independently), with both accounting for 73.9% of the total variation collectively. The strong explanatory power indicates that environmental selection and biotic interactions are paramount [31,70,72]. Both NMDS clustering and PERMANOVA analysis consistently demonstrated that geographical location and cold seep type (representing distinct geochemical conditions) exert a strong and significant influence on community structure. This pattern reflects the powerful selective effect of local habitat filtering on fungal taxa, resulting in significantly higher community similarity within the same geographical region or cold seep compared to similarities observed between cross-regional or cross-seep sites [19,73,74,75].

Moreover, the key driving role of biotic interactions emerged as another important finding. As revealed by Mantel’s test and enfit analysis, SRB possessing the *dsr* gene and ANME possessing the *mcr* gene were the strongest predictors of the fungal community structure, since metabolic functions are carried out by microorganisms (Figure 4C). It strongly suggests that the core energy metabolic pathway, which dictates energy generation in cold seep ecosystems, is a paramount force shaping the associated fungal community [21,76,77,78]. The abundance of these genes was significantly positively correlated with fungal abundance and strongly negatively correlated with sediment depth (Figure 3), indicating that the association between fungi and chemoautotrophic microorganisms is tightest in the shallow sediments where the AOM process is most active. Consequently, fungi are inferred to be more than just passive responders; they are tightly coupled to the chemoautotrophy-dominated energy and material flows, establishing a complex interaction network [78]. Fungi may function as decomposers utilizing organic matter produced by ANME/SRB consortia, or even engage in yet unknown mutualistic relationships [33,34]. This inter-species interaction is likely a critical ecological process determining the colonization and proliferation of specific fungal taxa in these unique habitats [33,79,80].

Stochastic processes: Evidence for the influence of stochastic processes on fungal community assembly is provided by the significant distance-decay relationship observed, where the Bray–Curtis similarity is significantly negatively correlated with geographical distance (*p* < 0.001) [81,82]. This robust pattern, often considered a hallmark of dispersal limitation, suggests that increasing geographical separation intensifies the effects of both dispersal constraints and ecological drift, thereby reducing community similarity. It consequently indicates that the dispersal capacity of fungi in the vast ocean is relatively limited, positioning geographical isolation as a vital constraining factor on community structure at the global scale [83,84,85].

Collectively, we propose that the assembly patterns of cold seep fungal communities are hierarchically structured, with the dominant ecological mechanism shifting across spatial scales. At the global scale, dispersal limitation serves as the dominant mechanism, where the vast geographical distances and physical barriers restrict fungal propagation, causing the community to exhibit distinct regional characteristics and the observed distance-decay pattern. Moving to the regional or habitat scale, environmental selection becomes crucial, as specific cold seep types and specialized local conditions act as filters, selecting for fungal taxa adapted to their unique geochemical profiles. Finally, at the local micro-environmental scale, biotic interactions emerge as the key drivers, with complex interactions involving critical functional microorganisms, such as ANME/SRB, ultimately governing the final composition and ecological dynamics of the fungal community.

### 4.3. Limitations of the Study

While our study reveals distinct biogeographical patterns of cold seep fungi, several limitations must be acknowledged. First, the heterogeneity in sample processing—ranging from DNA extraction kits to sequencing platforms across the original studies—may introduce technical bias, although we applied rigorous quality control and normalization (CPM) to mitigate these effects. Second, due to the inconsistent metadata reporting in public databases, physicochemical parameters (e.g., methane flux, porewater chemistry) were not available for all datasets, which constrained a full environmental correlation analysis for every site. Third, metagenomics relies on DNA, which indicates the genetic potential rather than metabolic activity. The presence of genes does not guarantee they are being expressed. Future studies integrating metatranscriptomics or metaproteomics are necessary to confirm the active roles of these fungi.

## 5. Conclusions

Through a global-scale metagenomic analysis, this study delineated the unique distribution patterns and complex assembly mechanisms of deep-sea cold seep fungal communities. Our findings confirm that these environments harbor rich fungal diversity, notably dominated by Ascomycota, particularly the unclassified *Pyronemataceae* and genera such as *Aspergillus* and *Candida*, and characterized by a substantial proportion of unknown “dark matter fungi”. The abundance and community composition of fungi exhibited significant spatial and habitat heterogeneity, suggesting a strong effect of environmental selection. Further analysis demonstrated that the assembly of the cold seep fungal community exhibits hierarchical multi-scale characteristics. At the global scale, assembly is primarily constrained by geographic distance and dispersal limitation. Conversely, local-scale community assembly is jointly determined by the influence of sediment depth and tight biological interactions with key chemosynthetic microorganisms, specifically ANME possessing the *mcr* gene and SRB possessing the *dsr* gene. These findings lay a critical foundation for a deeper understanding of the biogeography and ecological functions of fungi in extreme deep-sea ecosystems. Future studies should integrate functional research to thoroughly dissect the causal mechanisms underlying these correlations, particularly focusing on the specific interaction modes between fungi and ANME/SRB, to comprehensively elucidate the ecological role of fungi in the deep-sea ecosystems.

## Figures and Tables

**Table 1:** Correlation coefficients between fungal abundance, species diversity, and biotic and environmental variables.

Variables		All Samples		Arctic		Western Atlantic		Gulf of Mexico		Northeast Pacific		South China Sea	
		Species	CPM	Species	CPM	Species	CPM	Species	CPM	Species	CPM	Species	CPM
Spatial	Sediment depth	−0.339 ***	−0.328 ***	−0.697	−0.714	0.901 **	0.829 *	0.773 **	−0.448	−0.418	−0.385	0.074	−0.085
	Water depth	0.336 ***	0.393 ***	-	-	-	-	0.591 *	−0.012	-	-	−0.235 *	0.490 ***
Biotic	Prokaryotes	−0.003	0.551 ***	−0.153	0.324	−0.302	0.050	−0.105	0.643 *	0.071	0.537 *	0.043	0.622 ***
	ANME	−0.123	0.130	−0.704	0.044	−0.368	0.012	−0.651 *	0.467	0.776 ***	0.393	−0.220 *	−0.094
	SRB	−0.015	0.263 **	−0.091	0.159	−0.336	0.092	−0.306	0.656 *	0.797 ***	0.450 *	−0.412 ***	0.027
	*dsr*	0.141	0.431 ***	−0.843 *	0.301	−0.819 *	−0.482	−0.708 **	0.579 *	0.748 ***	0.535 *	−0.044	0.340 **
	*mcr*	0.153	0.404 ***	−0.929 **	−0.481	−0.472	−0.073	−0.742 **	0.655 *	0.684 **	0.518 *	−0.096	0.262 *
	Carbon fix	0.143	0.320 ***	−0.883 **	−0.217	−0.489	−0.069	−0.807 **	0.537 *	0.542 *	0.553 *	0.109	0.066
Physicochemical	CH4	-	-	-	-	-	-	-	-	-	-	−0.810 *	−0.934 **
	TC	-	-	-	-	-	-	-	-	0.424	0.507 *	-	-
	TN	-	-	-	-	-	-	-	-	−0.202	−0.351	−0.305 *	0.063
	TS	-	-	-	-	-	-	-	-	−0.145	0.189	-	-
	TIC	-	-	-	-	-	-	-	-	0.071	0.361	-	-
	TOC	-	-	-	-	-	-	-	-	0.318	0.192	−0.297	−0.361 *
	C/N	-	-	-	-	-	-	-	-	0.586 *	0.628 *	-	-
	CaCO_3_	-	-	-	-	-	-	-	-	0.076	0.363	-	-
	SO_4_^2-^	-	-	0.542	−0.680	−0.747	−0.822 *	-	-	-	-	0.823 *	0.959 ***

Note: Species and CPM (count per million reads) indicate fungal species diversity and abundance, respectively. Prokaryotes, ANME, SRB, *dsr*, *mcr* and carbon fix represent the abundance of prokaryotes, anaerobic methanotrophic archaea, sulfate-reducing bacteria, dissimilatory sulfite reductases gene, methyl-coenzyme M reductase gene, and core genes of carbon fixation pathways, respectively. Significance: *, *p* ≤ 0.05; **, *p* ≤ 0.01; ***, *p* ≤ 0.001.

## Data Availability

The SRA accession numbers for raw metagenomic data are listed in Table S1.

## Supplementary Materials

The following supporting information can be downloaded at: https://www.mdpi.com/article/10.3390/jof11120878/s1, Table S1. Geographic and environmental information for global cold seep sediment samples used in this study; Figure S1. Comparison of alpha diversity of fungal community on different cold seeps, geographic sites, and cold seep types. GoM, Gulf of Mexico; AtlanticW, Western Atlantic; PacificEN, Northeast Pacific; SCS, South China Sea. Figure S2. Highly differentiated fungal genera among different cold seep geographic sites (A) and types (B). Differential abundance analysis was performed using Linear Discriminant Analysis Effect Size (LEfSe). The length of the bar represents the linear discriminant analysis (LDA) score. Only genera with an LDA score greater than 4.0 and a significance level of *p* < 0.05 are displayed. ***, *p* < 0.001; **, *p* < 0.01; *, *p* < 0.05. GoM, Gulf of Mexico; AtlanticW, Western Atlantic; PacificEN, Northeast Pacific; SCS, South China Sea.
