# Supplementary material for: Global Distribution Patterns of Dark Matter Fungi in Cold Seep: A Metagenomic Meta-Analysis
Source: J Fungi (Basel). 2025 Dec 11;11(12):878. doi: 10.3390/jof11120878 (PMC12734366; doi:10.3390/jof11120878)
Supplement: Supplementary file 1 [file jof-11-00878-s001.zip › Supplementary files.pdf]

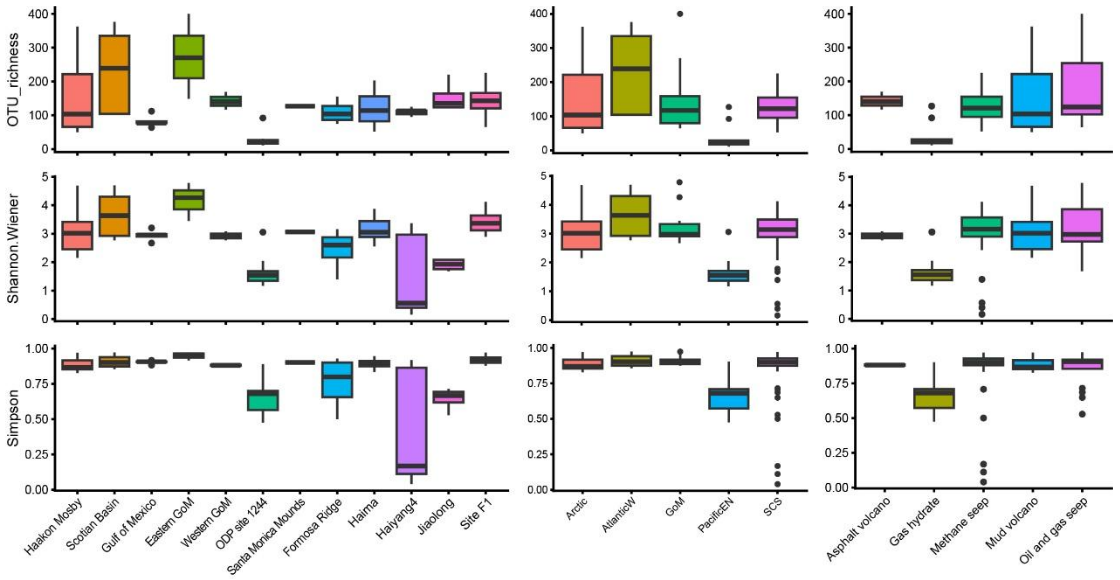

Figure S1. Comparison of alpha diversity of fungal community on different cold seep, geographic sites, and cold seep type. GoM, Gulf of Mexico; AtlanticW, Western Atlantic; PacificEN, Northeast Pacific; SCS, South China Sea.

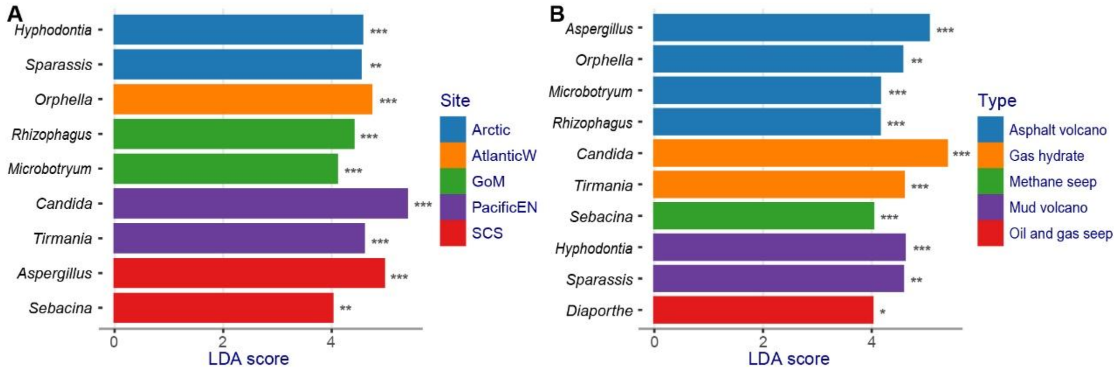

Figure S2. Highly differentiated fungal genera among different cold seep geographic sites (A) and types (B). Differential abundance analysis was performed using Linear Discriminant Analysis Effect Size (LEfSe). The length of the bar represents the linear discriminant analysis (LDA) score. Only genera with LDA score greater than 4.0 and a significance level of $P < 0.05$ are displayed. ***, $P < 0.001$; **, $P < 0.01$; *, $P < 0.05$. GoM, Gulf of Mexico; AtlanticW, Western Atlantic; PacificEN, Northeast Pacific; SCS, South China Sea.
